# Clinical and Genetic Characteristics of Splicing Variant in CYP27A1 in an Iranian Family with Cerebrotendinous xanthomatosis

**DOI:** 10.29252/ibj.25.2.132

**Published:** 2021-01-19

**Authors:** Zahra Rashvand, Kimia Kahrizi, Hossein Najmabadi, Reza Najafipour, Mir Davood Omrani

**Affiliations:** 1 Department of Medical Genetics, Shahid Beheshti University of Medical Sciences, Tehran, Iran;; 2 GeneticsResearchCenter, University of Social Welfare and Rehabilitation Sciences, Tehran, Iran;; 3 CellularandMolecularResearchCentre, Qazvin University of Medical Sciences, Qazvin, Iran

**Keywords:** Cerebrotendinous xanthomatosis, CYP27A1, Intellectual disability, Iran, Whole exome sequencing

## Abstract

**Background:**

CTX is a rare congenital lipid-storage disorder, leading to a progressive multisystem disease. CTX with autosomal recessive inheritance is caused by a defect in the *CYP27A1* gene. Chronic diarrhea, tendon xanthomas, neurologic impairment, and bilateral cataracts are common symptoms of the disease.

**Methods:**

Three affected siblings with an initial diagnosis of non-syndromic intellectual disability were recruited for further molecular investigations. To identify the possible genetic cause(s), WES was performed on the proband. Sanger sequencing was applied to confirm the final variant. The clinical and molecular genetic features of the three siblings from the new CTX family and other patients with the same mutations, as previously reported, were analyzed. The *CYP27A1* gene was also studied for the number of pathogenic variants and their location.

**Results:**

We found a homozygous splicing mutation, NM_000784: exon6: c.1184+1G>A, in *CYP27A1 *gene, which was confirmed by Sanger sequencing. Among the detected pathogenic variants, the splice site mutation had the highest prevalence, and the mutations were mostly found in exon 4.

**Conclusion:**

This study is the first to report the c.1184+1G>A mutation in Iran. Our findings highlight the other feature of the disease, which is the lack of relationship between phenotype and genotype. Due to nonspecific symptoms and delay in diagnosis, CYP27A1 genetic analysis should be the definitive method for CTX diagnosis.

## INTRODUCTION

Cerebrotendinous xanthomatosis is a rare autosomal recessive lipid storage disease caused by a defect in* CYP27A1 *gene, encoding the mitochondrial cytochrome P450 sterol 27-hydroxylase enzyme^[^^1^^]^. As a result of this deficiency, certain metabolites, such as cholesterol and cholestanol, accumulate in various tissues, particularly eye lenses and muscle tendons, as well as in the central nervous system^[^^2^^,^^3^^]^. CTX has neurologic and systemic presentations^[^^4^^-^^6^^]^, and its clinical symptoms include chronic diarrhea, cognitive impairment, intellectual disabilities, bilateral cataract, and tendon xanthomas. The clinical manifestation of CTX is highly heterogeneous, making CTX diagnosis difficult in the early stages^[^^7^^,^^8^^]^. In this sense, the molecular detection of *CYP27A1 *mutations improves the robustness of diagnosis.

CTX is more prevalent among female than male individuals (~55% vs. 45%) worldwide^[^^9^^]^. Although CTX is considered as a rare disorder, it may be less commonly diagnosed because it overlaps with other conditions with nonspecific symptoms^[^^10^^,^^11^^]^. There is noticeable variability in the clinical presentations, severity, and age of onset among patients^[^^9^^]^. The prevalence of this disease in our country is unknown; we had only one case report without molecular diagnosis and one case with c.1476+2T>C mutation^[^^12^^]^.

In the present study, we described three CTX siblings with c.1184+1G>A mutation in *CYP27A1 *gene, which is reported for the first time in Iran. This family is biologically unrelated but belongs to the same ethnic group. Such evidence indicates that the prevalence of *CYP27A1* variant in this particular population is likely high and reminds the importance of carrier screening. The clinical and molecular features of CTX patients were analyzed and compared with other patients with the same mutation. Also, the *CYP27A1* gene was analyzed for the number of pathogenic variants and their location.

**Patients’ data**

***Index family***

**Fig. 1:**
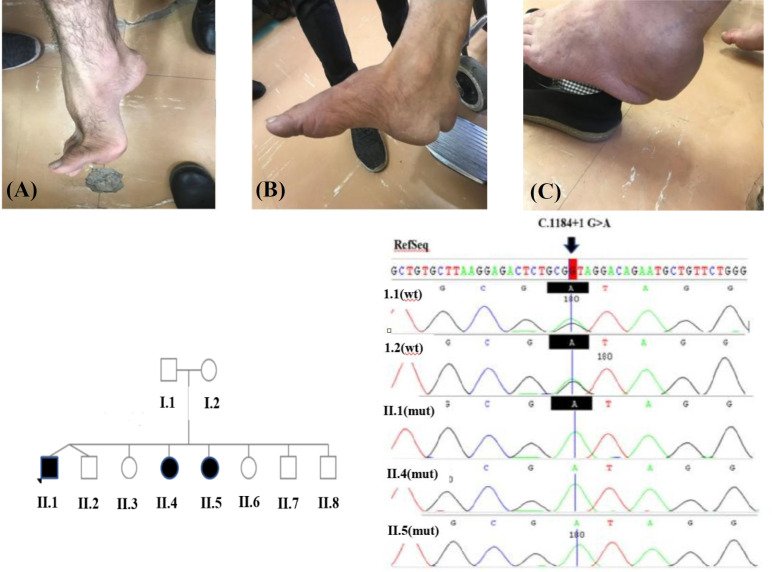
Pes cavus deformity and Tendon xanthomas of the patients aged (A) II.1: 38, (B) II.4: 51, and (C) II.5: 45, together with the pedigree of family and Sanger sequencing of the *CYP27A1 *variant in the family. Wild type (wt) and mutated (mut) sequences; Arrow shows the mutated position

This study recruited a non-consanguineous family from the same origin, with three affected children, a boy (proband) and two girls aged 38, 45, and 51 years, respectively (Fig. 1). Parents were not relatives but from the same ethnic group. The clinical symptoms of the proband and her siblings are listed in Table 1.

**Case no. 1 (II.1)**

The 38-year-old male was proband and the result of a dizygotic twin normal pregnancy and delivery. The mother did not report a history of an accident or infection during pregnancy, and the twin brother was healthy. The proband initially had the history of seizures 15 years ago, which then repeated every four to five years. He also had a history of cataract in early childhood, which was operated a year ago and had normal psychomotor development during his childhood. At school, due to the lack of academic achievement, intellectual disability was diagnosed. At age 27, he was found to have tendinous masses at the metacarpophalangeal joint, which was operated five years ago. The muscle strength was satisfactory, but according to the patient’s report for the past two years, it had been weakening, accompanied by the loss of power in muscles. Upon admission, neurological examination revealed ataxia, mood change, and cognitive decline. Also, he had hemorrhoid surgery last year.

**Table 1:** Clinical characteristics and molecular genetic features of CTX patients with c.1184+1G>A

**Type of mutation**	**Homozygous**				**Heterozygous**				
**Case/gender**	**CaseII-1/M**	**CaseII-4/F**	**CaseII-5/F**	**Verrips** ***et al.*** ^[^^9^^]^ ***/*** **NA**	**Garuti** ***et al.*** ^[^^13^^]^ ***/*** **F**	**Lee ** ***et al.*** **/NA** ^[^^14^^]^		
						**Case** **800-3**	**Case** **1000-3**	**Case** **1100-3**
Age (y) at onset	6	5	6	10	7	NA	NA	NA
Age (y) at diagnosis	38	51	45	36	45	NA	NA	NA
Age (y) at present	40	53	47	NA	NA	NA	NA	NA
Intellectual disability	+	+	+	+	+	NA	NA	NA
Tendon xanthomas	+	+	+	+	+	+	+	+
Juvenile cataracts	+	+	+	+	+	+	-	-
Foot deformity	+	-	-	NA	NA	NA	NA	NA
Chronic diarrhea	+	+	+	-	-	NA	NA	NA
Ataxia	+	+	+	+	-	+	+	+
Spastic paraparesis	+	+	+	+	+	+	+	+
Epilepsy	-	-	-	-	NA	NA	NA	NA
Parkinsonism	-	-	-	-	NA	NA	NA	NA
Peripheral neuropathy	+	+	+	+	NA	-	+	-
Seizures	+	-	-	NA	NA	NA	NA	NA

M, male; F, female; NA, not available

**Case no. 2 (II.4)**

The 51-year-old female was older sister of the proband. She was the result of a normal pregnancy and delivery. The mother did not report a history of an accident or infection during pregnancy. She also had normal psychomotor development during her childhood, and like her brother she was diagnosed with intellectual disability at school. She always had diarrhea. At age 30, she had an operation on cataract, and at age 35, she had a tendinous mass in the tendon of the Achilles, left knee, and finger joints. In addition, at age 45, she underwent another surgery for a blood clot on the brain. Added to these was a laser surgery carried out later to open up gallbladder outflow obstruction caused by gallstone. Three years ago, in 2017, she lost the ability to walk so that she needed help to get up and walk. She had experienced no seizures in her lifetime. At examination, her deep tendon reflexes were absent, and her muscle power was too weak to walk. She also had poor communication with those around her and suffered from ataxia and mood change.

**Case no. 3 (II.5)**

The 45-year-old female is the second sister of the proband. Like her brother and sister, she was the result of a normal pregnancy and delivery with no history of infection or accident during these periods. She also had normal psychomotor development and detected with intellectual disability at school. At age 30, she had a cataract surgery. Also, she underwent another surgery about six years ago because of the tendinous mass in the tendon of the Achilles. Like her sister, she had lost the ability to walk two years ago and could only get up and walk with help. She had no history of seizures but suffered from ataxia, which became more severe over time. At examination, she had ataxia, decreased muscle strength, and mood change.

## MATERIALS AND METHODS

The present study was conducted in Shahid Beheshti University of Medical Sciences (SBUMS), Tehran, Iran in collaboration with Qazvin University of Medical Sciences, and University of Social Welfare and Rehabilitation Sciences, Qazvin, Iran. A non-consanguine family with three affected siblings with non-syndromic intellectual disability was recruited for further molecular investigation. The parents were from the same village in Qazvin Province, the northwest of Iran. To prepare participants for the complete clinical examination and genetic counseling, we first collected 10 ml of whole blood from each patient, parents, and unaffected siblings in EDTA anticoagulant. Thereafter, genomic DNA was extracted according to the standard procedures^[^^15^^]^. The DNA quality was evaluated by electrophorese on 1% agarose gel and quantified using Nanodrop2000C Spectrophotometer (Thermo Fisher Scientiﬁc, Waltham, MA, USA), The families had previously been excluded from chromosomal abnormalities (by karyotyping), Fragile X syndrome (by molecular methods or Southern blotting), and metabolic diseases (by tandem mass spectrometry).

**WES**

The affected boy (proband) was selected to perform whole-exome sequencing. DNA library preparation, DNA target enrichment, and capture were carried out by the Agilent SureSelectXT2 kit (version 6; Agilent Technologies, Inc., Santa Clara, CA, USA). Finally, the Illumina NextSeq 500 system (Illumina Inc., San Diego, CA, USA) was used for the exome-sequencing, which uses 101-bp paired-end read with the mean depth of coverage 53× with 95.4% and 91.2% coverage at 10× and 20×, respectively. WES was analyzed according to our previous publication^[^^12^^]^. In summary, FastQC 11.5 software (https://www.bioinformatics. babraham.ac.uk/projects/fastqc/) was applied to check and confirm the quality of the sequences and also Burrows–Wheeler Aligner (version 0.7.12-r1039) was employed for aligning the raw reads with the reference genome (human genome 19 version, Genome Reference Consortium GRCh37)^[^^16^^]^. The Picard toolkit (https://broadinstitute.github.io/picard/) and GATK package^[^^17^^]^ were used for trimming, filtering, base recalibration, coverage determination, and insertion/deletion realignment of the Sequence Alignment Map files, and then variants were called using the Unified Genotyper module of the GATK package. Variants with a frequency below 1% were selected based on different human population databases (1000 Genome project; http://www.1000 genomes.org), Genome Aggregation Database )genomAD; https://gnomad.broadinstitute.org/), ExAC (http://exac.broadinstitute.org/), ESP6500 (http://evs. gs.washington.edu/EVS/) and in-house database of 800 Iranian control samples published in Iranome (http://www.iranome.com/). Then synonymous variants located outside the boundaries of 10 bp from the exons, and all non-coding regions were excluded. In the next step, variants belonging to the known genes in ID/DD^[^^12^^,^^18^^]^ were first considered, and then the pathogenicity of nonsynonymous, indels, deletions, and splice-site variants were examined. Besides, compound heterozygous/ homozygous variants were checked by *in silico* prediction algorithms, including Mutation Taster^[^^19^^]^, CADD (https://cadd.gs. washington.edu/) and PROVEAN (http://provean.jcvi. org/index. php)^[^^20^^]^, as well as *in silico* nucleotide conservation from GERP scores^[^^21^^]^. Finally, the selected variants were reviewed in OMIM database, ClinVar database (https://www.ncbi.nlm.nih.gov/ clinvar/), and locus/disease specific databases and evaluated for the relationship between phenotype and genotype and the causative agent of the disease. The final variant was confirmed by conventional Sanger sequencing. Co-segregation analysis was performed among family members, and variant analysis was carried out according to the patient's ethnicity.

**Ethical statement**

Methods for patient data and sample collection was approved by the Research Ethics Committee of Shahid Beheshti University of Medical Sciences, Tehran, Iran (ethical code: IR.SBMU.MSP.REC.1398.798). Written informed consents were obtained from the parents of the three patients for molecular analysis and any patient images. 

## RESULTS

 We identified a homozygous splicing mutation, NM_000784: exon6: c.1184+1G>A in *CYP27A1 *gene (Table 2). The G>A transition could impair RNA splicing and led to the generation of premature stop codon in mRNA and finally produced three abnormal transcripts. Premature stop codon activated the mechanism of nonsense mediated decay and caused undetectable levels of mRNA in patient fibroblasts. This mutation inherited in an autosomal recessive pattern and in population databases (genomAD, ExAc, ESP6500, 1000 Genome Project, and Iranome (Iranian database: www.iranome.com) was not found. Sanger sequencing confirmed homozygous pathogenic all in the proband and heterozygous status in the parents. The variant was not present in above-mentioned population databases and reported to be pathogenic in ClinVar with Variation ID: 65833. 

**Table 2:** Characteristics of mutation c.1184+1G>A in* CYP27A1* gene

**Gene**	**Change** **DNA**	**Change** **Protein**	**SIFT_pred**	**MutationTaster**	**FATHMM-** **MKL**	**CADD**	**DANN**	**GERP** **++ RS**	**ACMG**
*CYP27A1*	NM_000784: exon6: c.1184+1G>A	Splicing	D	Disease causing	Damaging	27.3	0.9954	5.4899	PVS1, PM2, PP3, PP5, pp1

SIFT_pred (SIFT predictions), a SIFT score predicts whether an amino acid substitution affects protein function. The SIFT score ranges from 0.0 (deleterious) to 1.0 (tolerated); D, deleterious; MutationTaster, a free web-based application to evaluate DNA sequence variants for their disease-causing potential; FATHMM-MKL, a high-throughput web-server capable of predicting the functional consequences of both coding variants, i.e. non-synonymous single nucleotide variants and non-coding variants in the human genome. DANN, a deep learning approach for annotating the pathogenicity of genetic variant

## DISCUSSION

The *CYP27A1 *gene, with a length of about 18.6 kb of DNA, is located on the long arm of chromosome 2(2q35) and consisted of nine exons and eight introns^[^^22^^]^. The largest transcript is about 1895 bp long and encodes Sterol 27-hydroxylase, an enzyme protein with 531 amino acids. The mature enzyme contains 498 amino acids and carries a 33-amino acid mitochondrial signal sequence^[^^22^^,^^23^^]^. Sterol 27-hydroxylase belongs to the mitochondrial cytochrome P450 family catalyzing the initial oxidation of the side chain of sterol intermediates during the hepatic bile acid synthesis^[^^24^^,^^25^^]^. Adrenodoxin-binding site (residues 351–365) and the heme-binding site (residues 435–464) are two highly protected regions in the mature enzyme and interact with adrenodoxin and adrenodoxin reductase^[^^22^^,^^23^^,^^26^^]^. Sterol 27-hydroxylase can also hydroxylate vitamin D3 at the C-1 and C-25 positions^[^^25^^]^.

*CYP27A1* is expressed in various tissues with the highest expression in the liver and then in the lung, followed by ileum and brain^[^^27^^]^. Defect in *CYP27A1 *gene causes a very rare disease, CTX. The clinical presentation of CTX is initially slow and then progressive process, including neurological and non-neurological symptoms. Some of these symptoms occur in childhood or adolescence and some in adulthood. The first clinical symptom to develop is neonatal cholestatic jaundice, which has been reported in numerous cases^[^^28^^]^. Other symptoms that begin in infancy or childhood are chronic diarrhea, bilateral cataracts, and xanthoma. Tendon xanthomas usually occur in the Achilles tendon, fingers, elbows, and knees as well as in the brain^[^^6^^,^^9^^,^^27^^,^^29^^]^. Neurological presentations are the most debilitating factor in this disease and are present in many cases at diagnosis. Intellectual disability and gait disorders reflecting neurologic dysfunction are common symptoms. Developmental delay, cognitive impairment, and learning difficulties are other neurological symptoms that may be developed during childhood^[^^9^^]^.

Currently, there are about 259 variants of *CYP27A1 *gene, of which about 85 variants are considered pathogenic or likely pathogenic. These variants are categorized as 14 frameshift (21.8%), 14 missense (21.8%), 17 nonsense (26%), and 19 splice site (29%) and by variant type: 15 are deletion (17%), 17 duplication (19%), 1 indel (1.1%), 3 insertion (3.4%), and 52 single nucleotide (59%). The distribution of these mutations in exons is as follows: exon 1 (7.8%), exon 2 (14%), exon 3 (15%), exon 4 (21%), exon 5 (3%), exon 6 (17%), exon 7 (10%), exon 8 (10%), and exon 9 (1.5%). Based on ClinVar database, this information indicates that the splice site mutation has the highest prevalence, and exon 4 has the highest amount of mutations.

The mutation c.1184+1G>A is located in the first nucleotide of intron 6. Its location at the intron-exon boundaries affects mRNA splicing, resulting in three abnormal transcripts. In the first transcript, by activating a cryptic donor splice site and skipping 89 bp of exon 6, the 5*' *half of exon 6 joins directly to exon 7. By changing the reading frame and creating a premature stop codon in this abnormal mRNA, a 348-amino acid truncated protein containing 16 new amino acids was formed at the end of its carboxy terminus. This truncated protein did not contain the three essential amino acids (Lys354, Lys358, and Arg362), which are supposed to play an important role in ferredoxin-binding domain. On the other hand, this shortened product, as iron–sulfur proteins, cannot be efficiently involved in a number of metabolic reactions in electron transfer^[^^30^^]^. Also, this truncated protein lacks the heme-binding domain^[^^30^^,^^31^^]^. In the second abnormal product, following the complete removal of exon 6, exon 5 is connected directly to exon 7, leading to a frameshift and generation of a premature stop codon. This abnormal mRNA appears to encode a 322-amino-acid protein that contains 16 new amino acids at the end of carboxy and lacks the ferredoxin and heme-binding domain. In the third abnormal mRNA, exons 6 and 7 are both completely removed, and exon 5 is connected to exon 8. This event does not alter the reading frame, but the translated product is predicted to be a protein lacking 82 amino acids (from Thr307 to Ans388) and having a total length of 422 amino acids. This deletion of the amino acid eliminates the ferrodoxin-binding domain, and the final truncated protein, although found in very small amounts, appears to be inactive. Garuti *et al.*^[^^13^^,^^32^^]^ checked the activity of this enzyme in patients with c.1184+1G>A mutation and observed that the sterol 27-hydroxylase activity is undetectable. This variant was first reported by Garuti *et al.*^[^^13^^]^ from Italy in 1997. It was reported to be heterozygous with another mutant allele in the patient indicated. Reported case was a 45-year-old female who at the age of seven developed symptoms such as cataracts, Tendon xanthomas, spastic paraparesis, mental deterioration, and psychiatric symptom, but no cerebellar signs like ataxia was observed. By comparing the symptoms of this patient with our patients, we found slight differences. For instance, ataxia is present in all of our patients, which gradually became more severe, but was not seen in the reported patient by Garuti *et al.*^[^^13^^]^. Another difference was that the c.1184+1G>A mutation has been reported as homozygous in our study and heterozygous in the study of Garuti *et al.*^[^^13^^]^. The variant c.1184+1G>A was also reported by Verrips *et al.*^[^^9^^] ^from Netherlands in 2000^[^^9^^]^. The patient manifested the initial symptoms at childhood and was then diagnosed with the disease at age 36. Cataracts, intellectual disability, cerebellar and pyramidal signs, as well as dementia and peripheral neuropathy were other related symptoms in this patient; however, diarrhea and epilepsy were not detected. The genotype of this patient was homozygous as our patients. The manifestations were almost similar, but diarrhea was present in all the three of our patients in the early years of life^[^^9^^]^. Another case was reported by Lee *et al.*^[^^14^^] ^in USA. The variant identified in that study was also heterozygote. These patients also had symptoms such as tendon xanthomas, cerebellar ataxia, and spastic paraparesis. Our patients also showed all these symptoms; however, the cataract, which was observed in all of our patients, was found in only one patient in the study of Lee *et al*.^[^^14^^]^. In our patients, there were phenotypic differences, though the genotype was quite similar. For instance, in case II.1, probands (male) had a history of seizures throughout their life, but no seizures were seen in female patients. In the proband, the pes cavus deformity was seen, but in female patients, the symptom was either absent or less severe. Moreover, the location of the xanthoma was present in the metacarpophalangeal joint in the male patient (proband), but in female patients, xanthoma was seen in the tendon of the Achilles. These discrepancies can possibly be due to dissimilarities in gender and genetic background. However, in this disease, there was phenotypic diversity between different patients, even those with the same genotype (sibling). All the clinical features of the cases with this variant are presented in the Table 1. Comparison of the symptoms in Table 1 also shows that individuals with the same mutation do not necessarily have similar phenotypes. In fact, there is no correlation between genotype and phenotype, which is another characteristic of this disease. In a study of CTX patients, Salen *et al*.^[^^7^^]^ found that foot deformity (pes cavus) was the most common symptom, followed by cataracts, spastic paraparesis, ataxia, dementia, tendon xanthomas, cognitive impairment and diarrhea, respectively. By comparing the incidence of these phenotypes with the patients listed in Table 1, we found that some of the symptoms, such as foot deformity, which is very common (100%), are not observed in all of our cases. Besides, cataract with an incidence of 88% and xanthoma with an incidence of 69% can be found in all of our cases. These results indicate that variable expressivity can be seen even in affected people with the same genotype in the family we reported.

CTX is one of the rare lipid storage diseases, and increasing awareness about this condition can improve the process of diagnosis and treatment. CTX is a disease often diagnosed in adulthood with a long delay (~20 years), from the onset of symptoms and diagnosis of the actual disease. Timely diagnosis and initiation of treatment at the early age play a significant role in preventing the development of some symptoms. In some countries, the prevalence of carriers is high, and accordingly screening programs are carefully implemented^[^^7^^]^. 

CTX is a less diagnosed metabolic disorder. In our country, only two cases of CTX with genetic diagnosis have been reported. This study will acquaint readers with the clinical and genetic features of CTX, since this disease has a good clinical response to treatment, early detection and genetic diagnosis can help reduce the complications of this disease because timely treatment prevents the accumulation of cholestenol in the tissues and the progression of the disease.
